# Assigning Function to Adult-Born Neurons: A Theoretical Framework for Characterizing Neural Manipulation of Learning

**DOI:** 10.3389/fnsys.2015.00182

**Published:** 2016-01-05

**Authors:** Sarah Hersman, Vanessa Rodriguez Barrera, Michael Fanselow

**Affiliations:** ^1^Department of Psychology, University of California, Los AngelesLos Angeles, CA, USA; ^2^Department of Psychiatry and Biobehavioral Sciences, University of California, Los AngelesLos Angeles, CA, USA

**Keywords:** neurogenesis, pattern separation, hippocampus, fear, learning

## Abstract

Neuroscientists are concerned with neural processes or computations, but these may not be directly observable. In the field of learning, a behavioral procedure is observed to lead to performance outcomes, but differing inferences on underlying internal processes can lead to difficulties in interpreting conflicting results. An example of this challenge is how many functions have been attributed to adult-born granule cells in the dentate gyrus. Some of these functions were suggested by computational models of the properties of these neurons, while others were hypothesized after manipulations of adult-born neurons resulted in changes to behavioral metrics. This review seeks to provide a framework, based in learning theory classification of behavioral procedures, of the processes that may be underlying behavioral results after manipulating procedure and observing performance. We propose that this framework can serve to clarify experimental findings on adult-born neurons as well as other classes of neural manipulations and their effects on behavior.

## Introduction

Behavioral neuroscience is by necessity an indirect assay of the function of neurons and neural networks using the direct measurement of behavior. Though, modern techniques allow direct manipulations of neuronal subclasses with great temporal and spatial precision, the readout of the effects of these manipulations are imprecise (e.g., calcium indicators), incomplete (e.g., electrophysiology), and/or indirect (e.g., fMRI, behavior). This imprecision in the tools used to measure these dependent variables necessitates a strong theoretical framework for designing and interpreting the outcomes of experimental manipulations. The case for assigning function(s) to adult-born neurons in the dentate gyrus is a perfect example of how muddy the experimental waters can become over a relatively well-defined question: what is the function of adult-born neurons in the dentate gyrus on learning and memory?

While not providing a single answer to this question, this review will seek to use behavioral studies that manipulated adult-born neurons, with an emphasis on fear conditioning, in order to demonstrate a terminological framework. This framework will categorize the types of manipulations or procedures conducted, the neural or computational processes hypothesized to be occurring during these manipulations, and the possible performance outcomes measured. This framework will be heavily based upon formal learning theory postulates, such as how animals learn about the valence of stimuli, how valence can spread to similar stimuli, and how the effects of neural manipulations may be different depending upon when the manipulation occurs, how animals are trained, and how they are tested.

Section Theoretical Traditions and Nomenclature will introduce the relationship between learning theory and computational modeling as it applies to the challenge of assigning function to adult-born neurons and pitfalls of an imprecise nomenclature. Section Classical Learning Theories of Generalization and Discrimination will review classical learning theories of generalization and discrimination, two important procedures for evaluating stimulus learning, and lead to discussion of our framework in Section A Framework for Constraining Procedure, Performance, and Process. Section Neural Substrates will review evidence for the neural substrates of these processes, including likely contributions of adult-born granule cells, based upon analysis using the framework. In Section Adult-born Neurons: Acquisition, Generalization, Differentiation, Pattern Separation, and Pattern Completion, we will specifically address the functions of this population in acquisition, generalization, differentiation, pattern separation, and pattern completion. Section Procedure and Process Interactions explains the framework in terms of interactions of procedures with their underlying processes, while Section Conclusion concludes with suggestions for implementing this framework for experimental design to clarify the function of adult-born neurons.

## Theoretical traditions and nomenclature

Adaptive behavior requires differential responding to stimuli based on past experience. Ideally after an experience with danger we will respond with defensive behavior to the cues that truly mean danger but not to nonthreatening cues. This ability to selectively respond to relevant cues and withhold responding to non-relevant cues is defined as differentiation. Differentiation is critical, as too much may lead to exposure to life-threatening situations, while too little can result in the inappropriate fear characteristic of anxiety disorders. Two more-or-less orthogonal approaches have addressed the issue of differentiation. One is associative learning theory, which ascribes differentiation, or the lack of it, to continuous changes in associative strength acquired through experience (Spence, 1936). Key concepts in this approach are behavioral generalization and differentiation (Mackintosh, 1974). Another derives from computational models based on neural architecture, specifically the hippocampus (Marr, 1970). The key concept in this approach relates to computations that disambiguate similar neural representations (pattern separation) but also those that allow re-instantiation of past patterns of neural activity from current activation of a part of the past pattern (pattern completion).

Recently much attention has been focused on these ideas. One reason is the emergence of technologies that allow us to directly manipulate the neurons putatively engaged in differentiation. Another is the interest in the adult-born population of dentate gyrus granule cells that result from post-natal neurogenesis. These adult-born neurons bear some relationship to both psychiatric disease and differentiation but that relationship is far from clear. At least some of this absence of clarity is driven by an inconsistent application of the nomenclature for differentiation. Is discrimination a procedure, a conceptual process or a behavioral outcome? Formally, pattern separation refers to a computational process but can a task be a pattern separation task? Dual use of the term may lead to a mistaken inference that differentiation on a “pattern separation task” necessarily implies the use of a pattern separation mechanism. Imprecise or intuitive use of this terminology is likely to hinder interpretation of the empirical data. Clarity for some of these terms can be found in classical experiments in which ideas such as generalization and differentiation were characterized.

## Classical learning theories of generalization and discrimination

### Generalization

The study of adaptive responding, under the headings of differentiation and generalization, is solidly rooted in associative learning theory. Associative learning theory emphasizes the associative connections acquired during two procedures: Pavlovian learning, where learning concerns the relationship between 2 or more stimuli, and instrumental conditioning, which concerns the relationship between responses and stimuli. Pavlovian learning is the basis for how a neutral stimulus (conditional stimulus, CS, e.g., a tone or a scented chamber) paired with an emotionally-relevant stimulus (unconditional stimulus, US, e.g., a mild foot shock) can come to generate a response that was not present before the association (conditional response, CR, e.g., freezing, fear). After training, the animal can be tested through presentation of the same stimulus or a different stimulus, in order to see how strong or how specific the learning of the association was to the circumstances of learning.

One experimental procedure that has been used to study this learning is a generalization test, which measures how much a subject responds to a novel stimulus that has never been reinforced as a result of previous training with a similar stimulus. This response is thought to be based upon the shared features or number of elements that are present in both the trained and novel stimulus (Spence, 1937; Hull, 1947; Mackintosh, 1974; Rescorla, 1976). The behavioral outcome of generalization is observed if an animal exhibits a high degree of responding to the novel cue, treating it as if it were the original trained stimulus; differentiation is seen if this is not the case. Generalization or differentiation seem to be primary or spontaneous processes that may occur upon presentation of the novel stimulus at test, rather than a manipulation during training. An early study of generalization demonstrated the effect in pigeons, which were trained to peck at a vertical line or specific wavelength of light. The response of the pigeons was measured when that line was tilted to various degrees at test, or different wavelengths of light were given, creating a parabolic relationship between level of response and similarity of test stimulus, with the trained stimulus at the peak (Guttman and Kalish, 1956; Hanson, 1959).

More recent studies have investigated the generalization phenomenon within the context of fear conditioning. Rats and mice that are trained to fear a tone of a particular frequency show robust freezing responses to novel auditory stimuli that were never reinforced with shock (Duvarci et al., 2009; Quinn et al., 2009; Cushman et al., 2012). Some evidence suggests that generalization along simple sensory dimensions occurs upstream in primary and secondary sensory cortices, manifesting as increased receptive field size after conditioning. This is followed by decreased receptive field size after discrimination training, which also reduces behavioral generalization (Edeline and Weinberger, 1993; Chen et al., 2011). Other regions, such as the thalamo-amygdala pathway, may also mediate this generalization (Lennartz and Weinberger, 1992; Edeline et al., 1993), as fear generalization to novel tones is unaffected by auditory cortex lesions (Armony et al., 1997). However, other studies demonstrate an underlying hippocampal relationship to presumed simple sensory processes even though the hippocampus is expendable for a tone fear conditioning task (Kim and Fanselow, 1992). Quinn et al. (2009) paired a tone or a white noise (conditional stimulus, CS) with foot-shock (unconditional stimulus, US) and then made post-training NMDA lesions to the dorsal hippocampus. Rats were tested with both the trained stimulus and a novel auditory CS that had not been presented during training. All of the rats, with and without the hippocampal lesions, showed a normal freezing response to the trained cue. However, lesioned animals froze less to an untrained novel cue compared to sham controls. Importantly, this reduction in freezing to novel cues was not present for a separate group of rats that underwent unpaired CS-US training, suggesting a true associative effect.

Generalization of responding to context or other complex stimuli may be accounted for with sensory models, where the similarity of trained and tested stimuli is determined by the number of altered stimuli. Though object category fear generalization (e.g., animals, tools) has been demonstrated in humans (Dunsmoor and LaBar, 2013), the cortical changes underlying fear learning about these objects are the result of extensive and repeated prior experiences with the categories, and as such may be a distinct process than that underlying context fear generalization in rodents, which may occur after a single learning episode. Contextual fear learning is mediated by the hippocampus under normal conditions (Kim and Fanselow, 1992), while evidence suggests that context generalization may be at least partly under the control of extrahippocampal circuitry, as infralimbic lesions lead to increased context generalization (Zelikowsky et al., 2013) and nucleus reuniens may modulate prefrontal inputs to hippocampus to increase or decrease generalization (Xu and Südhof, 2013).

Context generalization in particular has much in common with the modern computational principle of pattern completion. This is the notion that after stimulus elements have been stored as a complete representation, only a partial set of stimuli should be sufficient for recall of the entire stored representation (Rolls, 2013). Context generalization is a result of how animals sample successive stimulus elements in their environments, and must decide at each moment if they are in a familiar environment or a novel one based upon their initially limited sensory dataset. Successful recall occurs when a few elements of a familiar context are sufficient for the animal to recognize that context. Context generalization occurs when one or more elements of a familiar context overlap with a novel context, and the animal incorrectly assumes that the novel context is the familiar one. In both cases, pattern completion leads to “identification” of the familiar context. The likelihood that pattern completion will lead to context generalization will relate to the number of stimulus elements sampled in the original context, the number of stimulus elements sampled in the novel context, intensity of paired stimuli with the original context, and likely other factors of the learning and recall experiences.

### Discrimination and differentiation

A second experimental approach to studying responding is discrimination, which is the procedure by which one stimulus is explicitly paired with a US (CS+) and another is explicitly unpaired (CS−). If successful, then after several repetitions of these conjunctions, the animal learns differentiation, responding specifically to the paired stimulus and withholding responses to the unpaired stimulus. This type of differentiation may take substantial experience. Unlike generalization, which may be entirely related to perception processes, the differentiation caused by discrimination procedures additionally depends upon learning/memory rather than simply perception. Therefore, discrimination procedures provide a valuable tool for assaying processes underlying learning-related performance (e.g., Riley, 1968).

Learning theorists have offered potential processes that occur during a generalization test or discrimination learning. In his research with fear conditioning, Rescorla and his colleagues found that during associative learning, the extent of generalization depends upon the degree to which the shared or overlapping elements have gained associative strength. This relationship between conditioning of overlapping elements and extent of generalization was also formalized into a mathematical model of Pavlovian and instrumental learning (Estes, 1950). Along these lines, contexts, in this case the sensory representation of the box in which the shock occurs, are treated as groups of stimuli rather than single functional units when assaying generalization (Rescorla, 1999).

Discrimination learning has been well-characterized by learning theorists. Each trial of CS+ and CS− can be regarded as conditioning and extinction trials, respectively (Spence, 1936, 1937). During CS+, excitation increases to the stimuli paired with the US. During CS−, initially excitation from CS+ generalizes to CS−, due to the overlapping elements. Later in discrimination learning, overlapping elements undergo extinction due to an inconsistent relationship with the US, while non-overlapping elements develop the ability to inhibit responding (i.e., conditioned inhibition), and behavioral responding decreases. Therefore, if the CS+ and CS− have common features there will be generalization of excitation and generalization of inhibition to each of the stimuli. At each point during training, the level of responding to each stimulus is a joint function of inhibition and excitation of that stimulus (Jenkins, 1965). Because this theoretical approach assumes stimuli are made up of many elements that each undergo continuous changes in associative strength over trials this class of models are referred to as Elemental-Continuity Theories of discrimination learning (Spence, 1936; Rudy and Wagner, 1975). This type of model, whereby discrimination training between similar stimuli has the effect of reducing conditioning of common elements, and increasing conditioning of unique elements, has been supported by experimental manipulations (Rescorla, 1976).

An alternative theoretical approach to discrimination looks at stimuli in a more holistic manner. This approach suggests that what is learned is more concept or rule based. For example animals may learn about “classes” of stimuli, and use rules to categorize stimuli as exemplars or non-exemplars of that class. For example, instead of one CS+ stimulus and one CS− stimulus, (Herrnstein et al., 1976) reinforced stimuli that had in common a “concept,” such as the presence or absence of an individual person. Pigeons successfully learned to respond to the correct concept, with similar error frequency for previously-viewed images as for novel images, suggesting that after sufficient discrimination training, generalization to similar stimuli during testing on novel images can lead to successful categorization of new stimuli.

Herrnstein et al. (1976) specifically chose to use very complex stimuli because they would be challenging to interpret in terms of common stimuli that varied along some simple stimulus dimension. One can also use rules to categorize stimuli that vary along a single dimension. For example, animals can learn to respond to the larger or the darker of two stimuli. Kohler (1918/1938) trained chimpanzees and chickens with a dark colored circle as a CS− and a lighter circle as the CS+. Once the discrimination was learned the animals were given a choice between the CS+ and an even lighter stimulus. Rather than choosing the original CS+ during the test the animals tended to choose the lighter novel stimulus. This suggests that the animals learned a rule, choose the lighter stimulus, and then applied this rule to novel situations. This type of discrimination training and testing was named transposition after the way we easily recognize a melody when it is transposed to a different musical key. The relationship of notes to each other and not the absolute notes are what matters. Based on such findings Krechevsky (1932) proposed an explanation of differentiation that was radically different than Elemental-Continuity Theory. He suggested that animals learn nothing about the absolute features of the CS but rather simply test a rule and stick with that rule as long as it works. In the Kohler experiment the rule would be choose the lighter stimulus. If a rule failed too often, the animal would simply abandon that rule and try another. Krechevsky's model predicts abrupt changes in behavior as the animal shifts between hypotheses. It also does not decompose stimuli into elements but rather treats them as a more integrated configuration. Thus, his theory is a Configural-Noncontinuity Theory (Rudy and Wagner, 1975).

There is a very long experimental history attempting to determine which of these views (Elemental-Contiguity vs. Configural-Noncontinuity) better captures the data. While phenomena like transposition might seem most congruent with Configural-Noncontinuity Theory, in many circumstances Elemental-Continuity theory could predict the outcome as well. For example, if one trains a discrimination where there is overlap in features, there should be generalization of inhibition from the CS− to the CS+. Continuity Theory then predicts that peak responding will not be to the actual CS+ but rather to a stimulus that is more distal to the CS−. This phenomenon is called Peak Shift (Spence, 1937; Hanson, 1959). What emerges from this research is that animals can learn to differentiate using either type of strategy but that the actual arrangement of training and testing encourages one strategy over the other (Riley, 1968). So rule-based strategies and transposition occur when the animals is trained with simultaneous presentation of the CS+ and CS-such that they can be compared. But sequential presentation of stimuli encourages conditioning to the absolute features and peak shift as predicted by Elemental-Continuity Theories. Stimulus complexity likely plays a role. It may be difficult to differentiate complex stimuli made up of a very large number of features that vary amongst multiple dimensions using elemental strategies. In those cases Configural-Noncontinuity solutions may be adopted. One example of this is in the Herrnstein concept formation type of experiment. When images are difficult to decompose into simple features animals may adopt configural solutions (Cook et al., 1985). In support of this, Aust and Huber (2003) found that in pigeons trained to discriminate photographs containing humans from those that did not, classification was disrupted when the configuration of the human form was disrupted.

A similar pattern may occur in contextual fear conditioning. Contexts are made up of many features that vary amongst many dimensions and modalities (space, smell, lighting, sound, etc.). Each encounter with a context is likely to differ in what features are sampled and the rate and pattern of sampling. And different contexts are likely to contain many common features. Recognition of a familiar context and differentiation of a novel context may be far easier if the contexts were stored as configurations and not as a set of decomposable elements. This may explain why rodents need a period of exploration of a context before they can condition to it (Fanselow, 1986) and also why exploration needs to be of the assembled components and not the individual features (O'Reilly and Rudy, 2000).

Overall, the theories above serve to describe strategies animals may use to process stimuli and choose between similar or different stimuli during a behavioral task. In order to situate adult-born neurons, as well as other neural processing units, firmly within these traditions of behavioral design and behavioral interpretation, we propose a framework for designing and analyzing behavioral experiments.

## A framework for constraining procedure, performance, and process

The theoretical constructs in learning theory can be valuable constraints on experimental design seeking to determine the function of adult-born neurons. One impediment to progress on this topic is vague terminology, which can blur findings among different fields. The terms “pattern separation,” “discrimination,” “pattern completion,” and “generalization,” seem to have different definitions in the behavioral, neurological, and computational literature and are sometimes erroneously applied across disciplines. When attempting to determine how these rich patterns of behavior are generated, it is essential to limit our findings to the domain in which they apply, rather than assume they apply in other modes and levels of analysis. For example, computational definitions of pattern separation and behavioral discrimination learning have been used in similar manners even though one is a process and the other is a procedure. Clarification of these definitions is an important first step before substantive comparisons and developments can be made across fields, and other efforts to clarify some of these terms and their relationship to behavioral and neural measurements have recently been made (Hunsaker and Kesner, 2013; Fanselow et al., 2014).

Learning theory can be a solid guide throughout this clarification process. A potential theory can be broken into three domains: procedure, performance, and process. The intervening variable, process, can also have both computational and learning theory components. Here we propose to specify terminology relating to each of these domains during fear learning and recall procedures, in order to reduce some of the spread of terminology from one level to another. Though, some of the terminology and hypothesized neural correlates are specifically related to contextual fear conditioning in the examples below, this model is appropriate for other types of associative learning.

The procedure refers to how the experiment is constructed (e.g., operational definitions). It is how the environment (laboratory or real-world) changes and is the antecedent cause in a cause-effect relationship. Below, we have created a table with number of training stimuli (1 or 2) and type of stimulus test (familiar or novel).

After training occurs between a single stimulus and reinforcement, the stimulus may be directly tested with a stimulus test in order to determine level of learning, or a similar stimulus may be presented to determine specificity of learning. During a stimulus test^1^ (Table 1), responding to the previously reinforced stimulus is measured without reinforcement; this is the standard test of recall that gauges the strength of memory formation. In a generalization test^3^ (Table 1), the animal is exposed to a novel stimulus similar along one or more dimensions to the trained stimulus and responding to the new stimulus is measured.

**Table 1:** **Learning Procedures**.

**Test**	**Training**	
	**1 Stimulus**	**2 Stimuli**
**Familiar**	^1^Stimulus Test	^2^Discrimination
**Novel**	^3^Generalization Test	^4^Hybrid
**2 Stimuli**	−	^5^Transposition Test

*Procedure categories resulting from varying number and type of stimuli presented during training and test. Subjects are trained with either 1 stimulus or 2 stimuli and tested with a familiar stimulus (alone), a novel stimulus (alone), or two stimuli. The non-bolded terms in the central boxes are suggested names for testing procedures following particular training regimens*.

After training with one reinforced and one non-reinforced stimulus, testing both stimuli without reinforcement and measuring the difference in responding is known as a discrimination test^2^ (Table 1). Unlike the generalization test procedure, which is usually a single presentation of a novel stimulus, discrimination involves repeated interleaved presentations of CS+ and CS−, and resulting differentiation performance is due to explicit training rather than baseline similarity between stimuli. We create the term “hybrid”^4^ (Table 1) here to designate a fusion between discrimination and generalization procedures, wherein after training between these two stimuli a third, novel stimulus is also presented for a generalization test^3^ (Table 1). In this situation, the explicit training in the discrimination procedure is revealed to alter the nature of generalization between the trained stimuli and a novel stimulus, in a phenomenon known as peak shift (Hanson, 1959). Similarly, explicit training in discrimination can also be revealed by testing simultaneously with a trained stimulus (usually the CS+) and a novel stimulus, in a transposition test^5^ (Table 1). In this case, the contiguity of the stimuli present a “choice” to the subject, and direct comparisons between responding to each of the stimuli can be made.

Performance is the directly measureable behavioral output that is used to infer the degree of learning about the stimuli. Performance is the dependent variable or the consequence of the environmental manipulation and is thus the effect part of a cause-effect relationship.

Successful learning about the stimulus leads to acquisition^1^ (Table 2), or responding to CS+. If the degree of training, including the CS intensity, US intensity, or number of trials, is below the threshold of learning, acquisition failure will be observed. If acquisition succeeds, a novel stimulus may also be tested, and either generalization or spontaneous differentiation^3^ will be observed to the novel stimulus (Table 2). If two stimuli are differentially trained, the responding will either demonstrate trained differentiation^2^, where responding is differential between the stimuli, or indifference^2^, in which there are similar levels of responding to the two stimuli (Table 2). Even in cases of trained differentiation, responding to the CS− rarely disappears entirely, and in reality there is a range of responding from differentiation to indifference. Successful differentiation, when tested with a single novel stimulus, leads to peak shift^4^ (Table 2), in which responding is higher to a novel stimulus than to the CS+, because it is more different from the CS− than the trained CS+ is. If two stimuli are tested after discrimination learning of concurrent stimuli, transposition^5^ (Table 2) may be observed. Similar to peak shift, responding may be higher to a novel stimulus than the CS+ when they are concurrently presented, due to the novel stimulus differing from the CS+ in the same manner as the CS+ differed from the CS− (e.g., follow the rule to pick the lighter stimulus).

**Table 2:** **Learning performance outcomes**.

**Test**	**Training**	
	**1 Stimulus**	**2 Stimuli**
Familiar	^1^ Acquisition	^2^ Trained differentiation
	Acquisition failure	Indifference
Novel	^3^ Behavioral generalization	^4^ Peak shift
	Spontaneous differentiation	Peak shift failure
2 Stimuli	–	^5^ Transposition
		Transposition Failure

*Performance categories that result from successful learning or failure to learn about one or more stimuli. With the same training and testing regimens listed in Table 1, the non-bolded terms in the central boxes are suggested names for subjects' behavior at test*.

The process refers to the intervening variable (e.g., a brain process/mechanism) that leads from the procedural level to the performance level. Each box in Table 3 contains the learning theory terminology; some neural correlates are discussed below.

**Table 3:** **Learning processes**.

**Test**	**Training**	
	**1 Stimulus**	**2 Stimuli**
Familiar	^1^ Excitation	^2^ - Excitation determines S+ responding - The balance of Excitation (CS+) and Inhibition (CS−), related by their distance along sensory dimensions, determines S- responding
Novel	^3^ Excitation related by distance along sensory dimensions from CS+	^4^ The balance of Excitation (CS+) and Inhibition (CS−) related by novel stimulus distance from the two trained stimuli
2 Stimuli	−	^5^ Concurrent training leads to rule-based comparisons of simultaneous tested stimuli; rule application may be stronger than particular excitation to CS+

*Hypothesized mechanics for learning that may underlie observed performance outcomes. The non-bolded terms in the central boxes are suggested names for the processes underlying observed performance outcomes in Table 2 that may help to explain the outcomes observed*.

One theory of learning about stimuli states that performance after learning depends upon the relative strength of three theoretical variables: excitation provoked by trained stimulus (CS+), inhibition of trained stimulus (CS−), and similarity of test stimulus to trained stimulus or stimuli. The process that occurs during a stimulus test is just a representation of the excitation^1^ peak, from training, in the response (Table 3). During a generalization test, the process is the excitation level depending upon the “distance,”^3^ or similarity, to the trained stimulus, with more similar test stimuli generating a stronger excitation, and increased performance (Table 3). In discrimination, excitation of CS− depends upon both^2^ distance from CS+ excitation and strength of inhibition of CS- due to non-reinforced trials (Table 3). Thus, the behavioral process underlying behavioral differentiation differs depending upon whether a generalization or discrimination procedure is used. Consistent with this, silencing mature dentate granule cells impacts contextual fear generalization but not contextual fear discrimination (Nakashiba et al., 2012).

A third stimulus is mapped onto this relationship in hybrid^4^ (Table 3) in an attempt to reveal the changes in excitation and inhibition due to training in discrimination; responding to this stimulus is related to both the excitation of A and the inhibition of B, as well as the distance of this stimulus from both of the trained stimuli (CS+ and CS−). Transposition is also behavioral expression of the underlying changes in associative strength, and often leads to a preference for a novel stimulus over the CS+. This is often taken as evidence for rule-based learning^5^ (e.g., always pick the lighter stimulus) rather than learning about absolute stimulus intensities that predict reward, and is a direct result of concurrent presentation of stimuli during training (Table 3).

**Figure 1:**
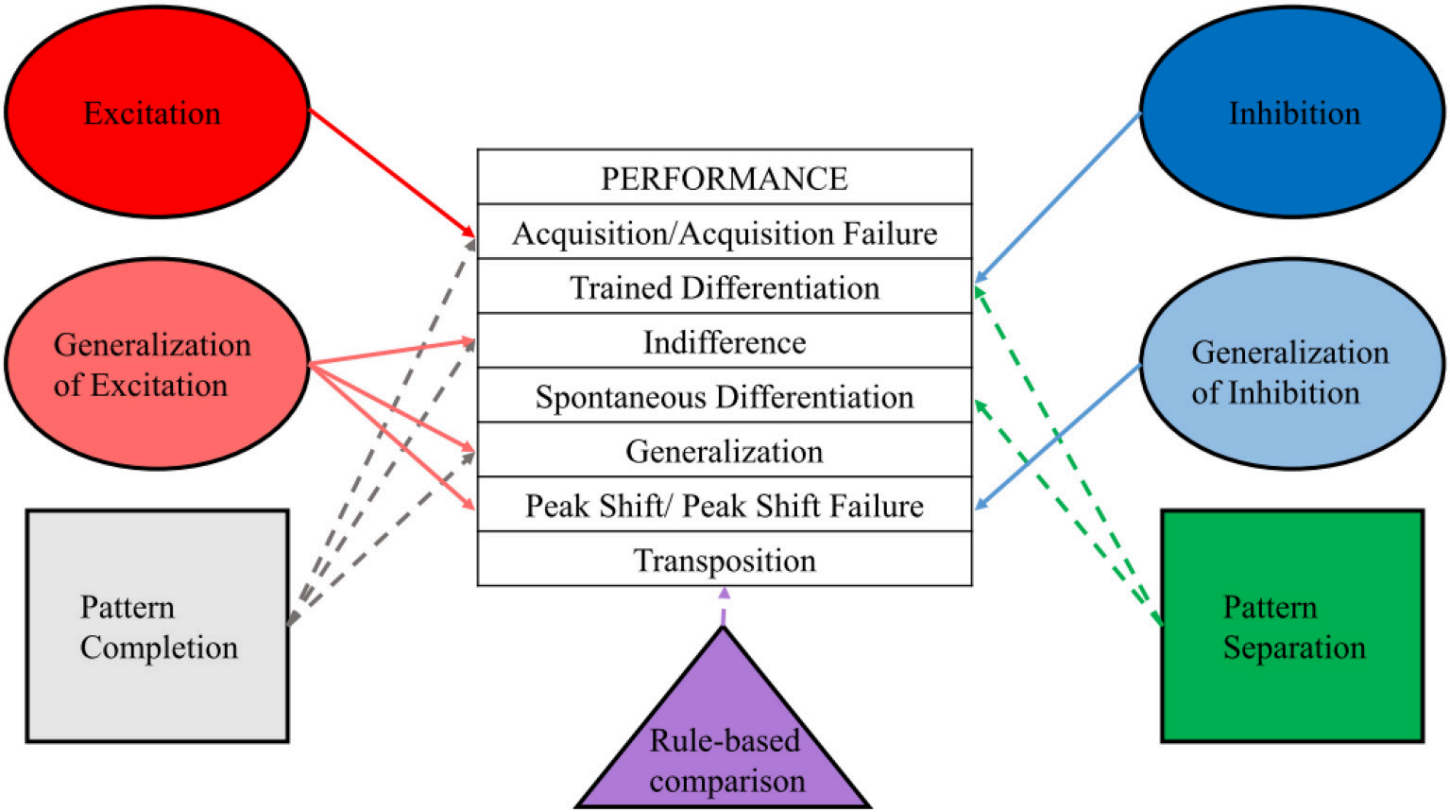
**Performance categories and underlying processes**. Differentiating procedure, performance, and process, and assigning a particular process or processes to each observed behavior will aid in interpretation of results and development of new tests of current hypotheses.

With this framework established (Figure 1), we now discuss the neural substrates that are thought to perform these processes, both from a computational perspective and as demonstrated by neural manipulations.

## Neural substrates

Though, the focus on this review is on hippocampal processing, and in particular the function of adult-born neurons in the dentate gyrus (DG), the hippocampus is but one structure embedded within a complex circuit with other regions (Bota et al., 2015) which also make contributions to the learning-theoretical constructs described above. These regions may provide processed sensory information to the hippocampal complex, aid in comparisons with past experiences, and exercise cognitive control over hippocampal processing. The mPFC has been shown to be involved with memory specificity, as it receives direct projections from hippocampal output regions and creates a closed loop with the hippocampus through projections through the nucleus reuniens (RE) of the thalamus (Vertes et al., 2007), a region implicated with other aspects of cognitive function and learning (Loureiro et al., 2012; Cassel et al., 2013; Cholvin et al., 2013; Varela et al., 2014). Furthermore, the mPFC is active during context memory acquisition, and inactivation of this region during acquisition leads to over-generalization of fear memory to a novel context (Xu and Südhof, 2013), so it may be playing a role in the degree of normal context generalization; this is likely due to the influence of the infralimbic cortex, as lesioning this region leads to strong generalization of fear between different environments (Zelikowsky et al., 2013). However, there are no direct projections between RE and the DG subunit of the hippocampal complex (Vertes et al., 2007), suggesting that the infralimbic to reuniens modulation of memory specificity may be complementary to this processing in the hippocampus. Two distinct pathways for altering memory specificity and generalization suggest that the degree to which generalization is seen at test may be due to an interaction between these two processes, though the degree to which each pathway exerts control during normal learning remains to be examined.

The amygdala is central to the discussion of fear conditioning and is the location of CS and US convergence for acquisition of fear (LeDoux, 1993). Under conditions of auditory fear conditioning, the basolateral complex (BLA) receives information from auditory cortex and also medial geniculate nucleus of the thalamus, creating a convergence of tone-shock inputs to build an association (Li et al., 1996; Swanson and Petrovich, 1998). Contextual fear conditioning requires hippocampal input to amygdala via the ventral angular bundle in order to build an integrated context representation, but context and shock association also occurs in the BLA (LeDoux, 1993; Maren and Fanselow, 1995; Fanselow, 2000). Though, our focus here is hippocampus, the importance of the amygdala cannot be excluded when making inferences about how computational and learning theory can be used to make predictions about underlying processes in fear learning.

The neural correlates of changes in associative strength to the CS+ and the CS- will differ depending upon the task parameters; however, differentiating these processes that result from particular training and testing parameters can guide the search for these distinct neural processes. In fear conditioning, excitation to the CS+ is thought to occur by strengthening of cortical and thalamic afferents to the basolateral amygdala. Inhibition is thought to require changes in projections from regions such as infralimbic cortex, while the stimulus “distance” is some function of the likelihood of multiple basolateral amygdala neurons responding to the same stimuli based on the similarity of their inputs (Fanselow et al., 2014). These three underlying processes make certain predictions about the effects of experimental manipulations. Decreasing excitability of the amygdala as a whole should lead to reduced excitation, potentially disrupted inhibition, but relatively little change in the overlap of amygdala neurons responding to multiple stimuli, as this is more a function of their common inputs than their activity levels. Alternatively, changing how different the CS+ and CS− are along one or more dimensions, or altering how different a novel stimulus is in a hybrid design, should significantly change the degree of overlap in amygdala representations of those stimuli, as well as behavioral responding to those stimuli.

Keeping in mind that this is not the only region in the brain in which these computations occur, we will now focus on the functional role of the hippocampal DG-CA3-CA1 trisynaptic circuitry, and on the function of adult-born neurons in this circuitry.

Computational models make predictions about hippocampal sub-regions and their relevance to aspects of differentiation. Theorists have grappled with a fundamental computational tradeoff in processing: if a region is specialized to store inputs effectively as unique memories, then it will be poor at retrieving those representations, and vice versa (Marr, 1970). For encoding, optimal characteristics include lower thresholds for plasticity and higher thresholds for reactivation of previously-encoded representations; the opposite would be optimal for recall. This trade-off seems to be avoided by having specific cellular populations that specialize in one or the other of the functions (Treves and Rolls, 1991; O'Reilly and McClelland, 1994; O'Reilly and Rudy, 2000; Rolls and Kesner, 2006; Nakashiba et al., 2012). Alternately, the hippocampus may enter into different computational states when stimuli are familiar or unfamiliar (Krasne et al., 2015).

In the hippocampus, the CA3 region has characteristics that improve its ability for memory recall based on cues that are presently available; namely, it contains a strong auto-associative network with a large number of recurrent collaterals among its principle pyramidal cells (Kesner et al., 2004; Kesner, 2013). The full generation of an initial representation can be accomplished with just a small portion of a pattern becoming active, such as with a partial cue during recall. This recall should even occur if the input is “noisy,” with some active units that were not in the original pattern. This computational principle is called pattern completion (O'Reilly and McClelland, 1994; O'Reilly and Rudy, 2000), and the importance of CA3 to the successful performance of pattern completion has some support from behavioral studies (Nakazawa et al., 2002; Nakashiba et al., 2012).

The dentate gyrus, which is the main input region to CA3, has characteristics that would make it effective at storing non-overlapping patterns: it has an extremely large number of granule cells in comparison to both its inputs (entorhinal cortex layer II) and outputs (CA3), as well as generally low firing rates and low number of granule cells that fire in any given environment (Jung and McNaughton, 1993; Chawla et al., 2005; Leutgeb et al., 2007), leading to the potential for an improved ability to store a large number of dissociable activity patterns within the synapses of the available cells. This computational principle of similar inputs being stored as dissimilar and dissociable outputs is called pattern separation, and is also attributed to the dentate gyrus by computational models (McNaughton and Morris, 1987; Treves and Rolls, 1992; Kesner, 2007; Treves et al., 2008).

An ability to separate similar inputs, such as different individuals in the example above, seems to be a prerequisite for responding to those inputs. Behavioral studies support a role for the DG in storing similar inputs, as DG lesions in rats cause impairments to the ability to discriminate between nearby food wells (Gilbert et al., 2001) and to remember which nearby arms have already been visited in the radial arm maze, with no effect on more distant arms (Lee and Solivan, 2010). By modeling the pattern completion function of CA3 and the pattern separation function of the DG, a clear picture emerges of a dynamic hippocampal circuit capable of rapid and non-overlapping storage during learning and cued reinstatement of stored representations during recall.

## Adult-born neurons: acquisition, generalization, differentiation, pattern separation, and pattern completion

The addition of adult neurogenesis to the circuit complicates the picture. Rather than containing a static population of cells, the DG has a continual birth of new neurons throughout the lifespan (Altman and Das, 1965; Cameron et al., 1993). The subgranular zone of the dentate gyrus contains progenitors that divide to produce new granule cells and this phenomenon has been shown to take place across species, including humans (Eriksson et al., 1998; Gould et al., 1999). As a consequence of the generation and maturation of new cells, there are always mature and immature granule cells present at any given point in the dentate gyrus. In addition to these adult-generated neurons there is actually another cohort of granule cells that is present at birth and derived from the prenatal period, constituting a third population in the dentate. Thus, we can think of the three sets of cells that make up the dentate gyrus as developmental, adult-generated mature, and adult-generated immature. The latter two populations can be grouped into the term adult-born neurons. Although, systematic investigations into the direct comparison of these three populations has not been made, there is some evidence to suggest there might be unique contributions from each (Wei et al., 2011; Cushman et al., 2012; Nakashiba et al., 2012; Tronel et al., 2015, but see Stone et al., 2011).

A lot of interest has been garnered specifically for adult-generated immature granule cells and their role in learning and memory. These neurons are not simply copies of their neighboring mature granule cells, but rather have a period of high excitation/inhibition balance (Marín-Burgin et al., 2012) and distinct membrane properties leading to a lower threshold for LTP (Schmidt-Hieber et al., 2004; Ge et al., 2007), when they are more easily excited than their mature counterparts, and participate in memory processes when they mature (Abrous et al., 2005; Ramirez-Amaya et al., 2006; Kee et al., 2007). Although, a majority of new cells undergo programmed cell death, the limited number of surviving cells become highly interconnected within the local circuitry. For example, mossy cells from the hilus are responsible for the first glutamatergic innervations and new granule cells also start to send their mossy fiber projections to CA3 within days after mitosis (Kempermann et al., 2008; Chancey et al., 2014), where they make functional glutamatergic synapses (Toni et al., 2008). The computational role that new neurons play in this circuit is difficult to predict. The addition of new processing units would seem to improve the differentiating function of the DG, as more unused units are available to decorrelate inputs. However, the increased excitability of these new neurons during development has been suggested to decrease their selectivity, as a lowered threshold may increase their responding to a variety of stimuli across contexts, leading to more similar outputs rather than dissimilar outputs (Marín-Burgin et al., 2012). Furthermore, the addition of these units varies dramatically with age, peaking during the juvenile period and steadily decreasing with age. It is as yet unclear how the time-varying increase in number of processing units may interact with the neuronal properties of these units to contribute to behavioral outcomes of generalization or differentiation, and to other processes in the DG and dorsal hippocampus as a whole (Aimone et al., 2006, 2011).

Many attempts have been made to integrate these new neurons into the function of the dentate and hippocampal circuit as a whole (for reviews: Aimone et al., 2006, 2009, 2011; Zhao et al., 2008; Deng et al., 2010; Barker et al., 2011; Sahay et al., 2011; Kim et al., 2012; Shors et al., 2012; Epp et al., 2013; Piatti et al., 2013), yet isolation of their function is challenging in a complex circuit whose overall function is not fully understood. The interaction of these distinct cellular classes may be critical to certain functions, rather than each class having a prescribed functional role on its own. However, based on the outcomes of multiple studies, it seems clear that newly-born neurons are involved in at least some types of hippocampal learning. Techniques to reduce or eradicate adult neurogenesis have been refined over the years, from pharmacological and irradiation methods to genetically-modified mice with reduced or absent adult neurogenesis (Goodman et al., 2010; Cushman et al., 2012). After a reduction in adult-born neurons, impairments have been seen in context fear conditioning, discrimination of similar contexts, trace conditioning, recall of long-term MWM memory, reversal learning, radial arm maze (selective to near arms), novel-object recognition, and general HPC-dependent memory (Saxe et al., 2006; Hernández-Rabaza et al., 2009; Kitamura et al., 2009; Drew et al., 2010; Burghardt et al., 2012; Denny et al., 2012; Pan et al., 2012; Shors et al., 2012). However, for almost every task listed above, other labs have found no impairment due to loss of adult-born neurons (Shors et al., 2002; Dupret et al., 2008; Zhang et al., 2008; Jaholkowski et al., 2009).

We will focus on the domain of cued and contextual fear conditioning and try to parse the behavioral effect of manipulations of dentate neurons according to the procedures in Table 1. This allows us to offer suggestions for improving tests of their contribution. We will also provide structure for the debate of adult-born neuron involvement in computational processes of pattern completion and/or pattern separation.

### Context fear acquisition

As described above, context fear acquisition is defined as pairing one or more shocks with exposure to a context and returning to that context to test levels of fear. This pairing may occur in a number of ways, with varied task difficulty and learning outcomes. Direct CS-US pairing, where the context exposure (CS) begins and then one or more foot shocks (US) occur, leads to high levels of learning, due to the strong contingency between the CS and US. This may be contrasted with the lack of learning when the US precedes the CS, termed the immediate shock deficit, which cannot be rescued by repeated immediate shocks (Landeira-Fernandez et al., 2006), as the contingency between the CS and US remains weak or even negative in these circumstances.

Many manipulations can affect levels of contextual fear. Hippocampal lesions after learning will erase previously-acquired contextual fear memories, while hippocampal lesions before learning will lead to memories that do not endure across time (Zelikowsky et al., 2013), demonstrating that contextual fear circuitry is capable of some degree of compensation after damage (Wiltgen et al., 2006; Fanselow, 2010). Levels of contextual fear may also be affected by the intensity of shock, the number of shocks, the amount of time spent in the context before the first shock, complexity of the context, and pre-exposure to the context (Fanselow, 1980, 1986, 1990; Fanselow and Tighe, 1988; Fanselow et al., 1993; Drew et al., 2010).

There is, as yet, no consensus on the involvement of adult-born neurons in this task. In addition to the variance in contextual fear protocols, experimental results may vary due to type of ablation, extent of ablation, species, and any interactions of these variables. However, there is evidence that task difficulty may be a determining factor. One way in which difficulty may be increased is by decreasing the number of shocks. Though some studies using multiple shocks have seen reduced levels of context freezing with knockdown of adult neurogenesis (Winocur et al., 2006; Saxe et al., 2007; Wojtowicz et al., 2008), the majority of studies only see impairment when a single shock (Hernández-Rabaza et al., 2009; Ko et al., 2009) or weak conditioning protocols are used (Imayoshi et al., 2008); these deficits are rescued with multiple shocks or pre-exposure (Drew et al., 2010), both of which reduce the cognitive load of the task. Further, studies did not see effects on multiple pairings with context fear conditioning (Shors et al., 2002; Dupret et al., 2008; Zhang et al., 2008; Jaholkowski et al., 2009; Cushman et al., 2012). A comparison may be made with the fact that multiple conditioning trials also rescue fear learning in hippocampal-lesioned animals to the levels of sham controls (Wiltgen et al., 2006), raising the possibility that compensatory fear circuitry may also play a role in rescuing learning deficits in irradiated animals (Fanselow, 2010; Zelikowsky et al., 2013).

In the context of the paradigm described in Tables 1–3, this reduced ability to learn from a single shock after loss of adult-born neurons has a likely culprit: the excitation peak, and hence the strength of the fear memory, is reduced following loss of new neurons. This is supported by the reported increased excitability of new neurons, which may lead to their lower threshold for memory storage with a weak association. The lack of effect with multiple trials is consistent with this hypothesis, or potentially with compensation by other structures (Zelikowsky et al., 2013).

One possibility that must be addressed is that the variability in effects on context fear is due to the simple acquisition task not being selectively sensitive to the processing of adult-born neurons in DG. If the broad tuning of adult-born neurons improves salience of the context, for example, then subtle differences in the complexity of context environments, salience of transport method to the context conditioning chamber, and the presence of simple, salient contextual cues would determine the necessity of adult-born neurons for sufficient contextual processing. Adult-born neurons may not be essential for context fear conditioning, but they may become crucial only when no other individual, salient cues are available to signal proper responding. Rather than continuing to perform contextual fear conditioning experiments and simply testing fear acquisition, concrete hypotheses about the unique functioning of adult-born neurons in DG must be specifically tested so that alternatives may be eliminated. Context fear conditioning may be a viable method for such rigorous testing, but more varied hypotheses can be tested using generalization and trained differentiation as metrics than in using acquisition alone.

### Fear generalization

Although, tone fear acquisition is not hippocampus-dependent, generalization of fear to auditory stimuli is thought to depend, at least in part, on hippocampal processing. As mentioned above, animals will readily show fear responses to tones never paired with shock after tone fear conditioning with other auditory stimuli and this fear generalization can be reduced by dorsal hippocampal lesions (Quinn et al., 2009). This generalization was caused by training as animals did not respond similarly to both stimuli when they were trained with an explicitly unpaired procedure. Furthermore, selective deletion of all post-natal neurogenesis using a conditional knockout mouse line lacking DNMT1 in GFAP+ positive cells actually causes greater generalization to novel auditory stimuli (Cushman et al., 2012), suggesting that new neurons normally limit the levels of auditory stimulus generalization.

However, other structures may also be involved in generalization. In tracking the time-to-lesion interval and relating it to degree of context fear generalization, Wiltgen and colleagues found that detailed, less generalizable memories are always hippocampus-dependent, whereas animals that generalized to a new context showed no impairment with hippocampal inactivation (Wiltgen et al., 2010). These data suggest that while impairments to the hippocampus may alter levels of generalization seen, some forms of generalization may take place outside the hippocampus (Xu and Südhof, 2013; Zelikowsky et al., 2013).

As generalization is thought to be determined by the width of excitatory gradients after learning and the proximity of the test stimulus to the training stimulus, many of the same factors must be taken into consideration when performing a generalization test as an acquisition test. The broad tuning of adult-born neurons may lead toward either more or less generalization depending upon the complexity of the contexts, intensity of the training, and the similarity of the trained and tested contexts. Again, concrete hypotheses can help to use these factors to test how these adult-born neurons work differently from their neighbors.

### Fear differentiation

The method that has most consistently revealed sensitivity to manipulations of adult-born neurons is contextual discrimination learning. In a context fear discrimination task, one context will be paired with one or more foot shocks, while another context will be unpaired, typically with three or more trials of each context (Fanselow, 1981). The magnitude of difference between the characteristics of the contexts may be varied to modulate task difficulty, as may the number of trials. Often, differentiation is measured and compared by using the rate of differentiation, or differential pre-shock freezing to the two contexts, rather than absolute levels achieved after learning. Differentiation of similar contexts during contextual fear discrimination has been shown to be impaired by irradiation-induced loss of adult neurogenesis (Winocur et al., 2006; Winocur and Moscovitch, 2011; Nakashiba et al., 2012), post-training ablation of adult-born neurons (Arruda-Carvalho et al., 2011), and inhibition of NMDA receptors in adult-born neurons through deletion of NR2B subunit (Kheirbek et al., 2012). Furthermore, differentiation is improved by increasing the survival number of adult-born granule cells (Sahay et al., 2011).

Adult-born neurons may indeed affect differentiation in certain situations, but this role may be modulated by task difficulty. Silencing mature dentate granule cells without affecting the immature cells improves differentiation when contexts are very similar, with no effect when the contexts are relatively distinct (Nakashiba et al., 2012), suggesting that immature granule cells might assist in learning to differentiate similar stimuli. However, when contexts are extremely similar, loss of post-natal neurogenesis can in some cases lead to an enhancement in differentiation (Cushman et al., 2012), suggesting that immature cells may be hindering learning in this case. Another recent finding showed that increasing neurogenesis by wheel-running after training actually results in worse context discrimination and ablating neurogenesis in transgenic mice enhanced context fear learning (Akers et al., 2014). It seems likely that both immature and mature granule cells play a role in differentiation. This integrated view is supported by the fact that NMDAR knock-outs in all granule cells in the dentate lead to the same impairments in learning to differentiate similar contexts as have been found in other studies to be a result of the loss of one or the other population (McHugh et al., 2007). Future, studies should seek to determine not simply the effects on learning after loss of neurogenesis, but rather the function of those adult-born neurons in relation to the function of the DG, and even hippocampal complex, as a whole.

### Pattern separation

Despite frequent usage of the term “pattern separation” in the neurogenesis literature, the definition as it relates to behavioral tasks remains unclear. The superficial similarity of pattern separation to the outcome of differentiation led to modification of this principle from its original definition, particularly in the domain of the function of adult-born neurons. It has since been applied in a variety of ways: contextual discrimination learning, radial arm maze performance, and even sensory discrimination between visual stimuli or odor cues are all taken as evidence of “pattern separation” (Kim and Sun, 2013). Despite the fact that the labeling of these tasks is based more on intuition than computational evidence, successful completion of the task is taken as evidence of successful pattern separation, while impairment or failure is evidence of impaired pattern separation. However, it is difficult to see a task that would not fall under this basic component of learning and memory; at some level, any task requiring encoding or even sensory processing requires “pattern separation.” By solely using this term with its computational definition, we propose the following two criteria for impairment on a task to be labeled as a pattern separation impairment. Manipulation must result in: (1) Task impairment specific to a requirement to differentiate similar inputs, while less similar inputs are not impaired and (2) Cellular representations involving task stimuli, either in hippocampus or elsewhere, are altered to lead to an increase in “overlap,” either in the identity of the units, or in the responding of those units.

When classifying a task as involving pattern separation, these essential limits on the definition are sometimes neglected. In a study by Holden et al. (2012), young and elderly human subjects performed better in a visual matching task at large distances between the visual stimuli than at small distances, and elderly participants were worse at all distances than younger participants. This was taken as evidence that pattern separation abilities declined with age, even though the age-related impairment applied to all distances tested, rather than just very small distances. After focal brain irradiation, which irreversibly halts the birth of new adult-born neurons, mice are selectively impaired at spatial discrimination of stimuli with small spatial separation (Clelland et al., 2009). This may be a pattern separation deficit, but further evidence, in the form of demonstrating increased overlap in the neural representations of nearby radial arm maze arms, is necessary to conclusively demonstrate this. The irreversible nature of the focal brain irradiation makes this type of evidence difficult to obtain.

Stronger evidence for the participation of immature adult-born neurons in pattern separation can be found in changes in rates of differentiation after manipulation. When mature granule cell neurotransmitter release is blocked, so that only immature neurons are fully functional, mice show an increased rate of differentiation learning, which was taken as evidence of improved pattern separation (Nakashiba et al., 2012). After a genetic manipulation to increase the number of new neurons that survive by twofold, animals have the same increase in learning rate, with the same explanation (Sahay et al., 2011). In a separate study in rats, increased rate of differentiation of nearby stimuli was demonstrated in a touchscreen task after running induced an increase in neurogenesis. No effect of running was seen in aged animals whose rates of neurogenesis did not increase after running (Creer et al., 2010). Furthermore, genetic ablation of progenitors in order to knockdown the production of adult-born neurons lead to deficits in differentiation, supporting the notion that adult-born neurons are involved in aspects of this learning (Tronel et al., 2012). In all these tasks, the evidence that these manipulations of adult-born neurons lead to the required increased overlap of cellular representations has not been conclusively demonstrated.

It seems clear that there is a hippocampal task-dependent change that is mediated by adult-born neurons. However, it might be too imprecise to call this a change in “pattern separation.” An ability to detect small changes in inputs is required for success at these tasks, but the increase in rate of learning might not be attributable to those same processes. In fact, one study in aged rats showed a negative correlation between classical pattern separation and successful differentiation (Marrone et al., 2011). In this study, aged rats with negligible levels of neurogenesis have improved pattern separation, as measured by a decrease in overlap of immediate early gene activation between two environments, or even repeated visits to the same environment, compared to control animals. This pattern separation increase was correlated with a declining ability for older animals to differentiate between the different contexts in a sequential spatial recognition task. One possibility for this disconnect is that adult-born neurons could control activity of mature granule neurons, and by disrupting immature cells, mature cell firing and pattern separation abilities are disrupted (Lacefield et al., 2012). Another possibility is that this disconnect may be due to a requirement for overlapping representations for normal differentiation in the dentate gyrus, through a rate remapping of a small active population rather than global remapping between populations (Leutgeb et al., 2007). Perhaps, then, adult-born neurons improve differentiation, but through improved rate remapping rather than the global remapping typically required for pattern separation. Future, experiments will be required to determine the validity of these explanations.

The necessity to show a specific impairment in differentiating similar inputs is further confounded by the fact that similar inputs are generally more difficult to differentiate. Therefore, an improvement or impairment due to manipulating neurogenesis levels may instead be due to changes in conflict resolution or inhibitory learning, processes with very different proposed mechanisms. Furthermore, it is unclear computationally how new neurons might even participate in classical pattern separation. The general addition of units to a system has been proposed as one possible mechanism, as increasing the number of free units would necessarily improve a systems-level ability to store similar inputs on non-overlapping units. However, as immature adult-born neurons are on the whole more excitable/broadly tuned than their mature counterparts (Wang et al., 2000; Schmidt-Hieber et al., 2004; Marín-Burgin et al., 2012), they would be more likely to respond indiscriminately to both of two similar stimuli, thereby decreasing the ability of the organism to differentiate between those stimuli (Aimone et al., 2009, 2011; Abrous and Wojtowicz, 2015). How these two properties of adult-born neurons relate to cellular representations in the dentate gyrus, as well as behavioral outcomes on procedures such as discrimination, requires further investigation.

### Pattern completion

Pattern completion is a computational principle that has been applied, in the hippocampal literature, to the ability to reinstate a cellular representation from partial or noisy cues. This reinstatement ability can be demonstrated in a multiple-layer computational network of the hippocampus, and especially of the recurrent collateral network in region CA3 (Rolls, 2007). However, dissociation of this principle from simple “recall” can be challenging. This process sounds much like recall, where a full memory is reinstated by a retrieval cue. However, recall is a behavior and pattern completion may or may not be a process underlying that behavior. Recall can stem from any of the processes described in Table 3.

One behavioral task that has been related to pattern completion is a direct test of one principle of pattern completion: the relationship between missing cues and recall. In a study by Nakazawa et al. (2002), mice lacking functional NMDA receptors in the CA3 region and control littermates were trained on the Morris Water Maze to navigate to a hidden platform using four extra-maze cues. When three of these cues were removed, CA3-impaired mice had a significantly reduced preference for the platform location, while controls were unaffected (Nakazawa et al., 2002). This study was an important demonstration of the requirement for a functional CA3 region in computational pattern completion; however, it did not dissociate between effects on encoding or recall, as the transgenic animals developed with impaired CA3 function. A second study focused on mature and immature cells in the dentate gyrus and tested contributions to pattern completion processes using pre-exposure-dependent contextual fear conditioning (Nakashiba et al., 2012). In this behavioral task, animals are pre-exposed to a context without shock and the following day are given a very brief experience in the context before receiving a foot shock. Mice with silenced mature granule cells were impaired in this task during acquisition test. These mice were also impaired at the recall of MWM acquisition in the partial cue condition. An impairment in acquisition of the pre-exposure task is thought to demonstrate an impairment at reinstatement of a full representation due to brief exposure to cues (Fanselow, 1990; Rudy and O'Reilly, 2001); there may be a similar process occurring during impaired recall of MWM acquisition. Interestingly, the loss of immature cells increases the amount of time required for context processing before the first shock for successful acquisition (Drew et al., 2010). These data suggest that both immature and adult populations of granule cells may be involved in processing and integrating complex stimuli. This neural processing may relate to the computational process of pattern completion, though more refined experimental designs that selectively manipulate these populations during acquisition or acquisition test under a variety of task conditions would provide greater support for this idea.

In the context of our terminology, it is important to distinguish the principle of pattern completion from a simple recall or a generalization test. Changes in a generalization test depend upon changes in encoding, as behavioral expression at test is thought to be related to changes in the shape of learned excitatory gradients during learning. Pattern completion, on the other hand, is a recall-specific phenomenon, as it relates to successful activation of learned representations despite noisy or incomplete input patterns. The impairments in MWM related above (Nakazawa et al., 2002) cannot be defined as pattern completion deficits, because the same impairments could have resulted from imprecise encoding of the context, which would not be revealed until cues were removed and the task became more difficult, and which would be unrelated to deficits in recall. In order to hypothesize that a given task deficit is related to disruptions to pattern completion processes, then, the manipulation must be absent during the learning period and present during the test period. New neurons have not been related to pattern completion hypotheses *per se* (but see Frankland et al., 2013), but have participated in related hypotheses of pattern integration across time. Due to their early property of hyper-excitability and to successive “waves” of populations that become excitable and then mature, these neurons could participate in encoding similarities between events that happen near to each other in time (within a “wave”), known as pattern integration, and could help to differentiate events that occur across time (between “waves”), as different populations of neurons would represent these events (Aimone et al., 2006). These distinct properties based upon separation of training events could contribute to contradictory effects of manipulations of adult-born neurons on hippocampal-dependent behavioral tests. Tests of this hypothesis will hopefully shed some light on this relationship between adult-born neurons and pattern integration.

## Procedure and process interactions

Learning theory and computational neuroscience can both inform the current behavioral data on adult-born neurons as well as lead to new hypotheses and experiments. The performance measures that may result from the five modes of training and testing of stimulus processing (stimulus test, generalization test, discrimination, hybrid, and transposition test) have distinct underlying properties in both the learning theoretical and computational domains. Excitation is involved in acquisition, while generalization of excitation underlies indifference, generalization, and peak shift. Inhibition is involved in trained differentiation, while generalization of inhibition is most obvious in peak shift. Pattern completion shares many characteristics of excitation and generalization of excitation, and may be involved in acquisition, indifference, and generalization, though this process is likely acting at the recall rather than the learning stage of these procedures to reinstate, appropriately or inappropriately, the original learning representation based on cues present during test. Pattern separation, on the other hand, is most evident in trained differentiation and spontaneous differentiation, though this process is likely acting at the learning rather than the recall stage of these procedures to reduce overlap between competing representations. Rule-based comparison is one description for the hypothesized processes underlying transposition, and may act at the learning stage, recall stage, or both experimental stages. Both peak shift and transposition are difficult to categorize using either exclusively pattern separation or pattern completion terminologies, though these behavioral outcomes are likely due to processes occurring at the time of learning, and revealed by the test stimuli and testing procedure.

This complex web of interactions between different theories of learning serves to illustrate the care that must be taken in interpretation of effects of adult-born neuron manipulation. If a group lacking neurogenesis has improved differentiation (Sahay et al., 2011, for example), this could be due to either a sharpening of inhibitory gradients during learning or an increase in pattern separation capability. Increased generalization (Cushman et al., 2012), on the other hand, could be due to enhanced excitatory gradients or to overactive pattern completion mechanisms. It can be nearly impossible to dissociate the two effects from a behavioral result alone, necessitating the concurrent use of neuronal recording and cellular imaging technologies in order to examine the process-level changes that lead to the performance effect.

One should also be careful about making inferences about a computational process from a brain manipulation alone. While studies showing indifference between stimuli following a dentate manipulation will often invoke an explanation in terms of pattern separation, the same behavioral finding will rarely evoke a description of pattern separation when a region outside the hippocampus, such as prefrontal cortex, has been manipulated. Furthermore, if the dentate gyrus does contribute to pattern separation processes in certain circumstances, it certainly does not have a monopoly on this process. For example, there is some evidence that the olfactory bulb, classified as primary sensory cortex, participates in this process (Sahay et al., 2011), and other cortical and subcortical regions may prove to be involved in certain circumstances (Gilbert and Kesner, 2002).

As we begin to probe how adult-born neurons contribute to the process level of these procedures, and seek deeper understanding of associative learning networks as a whole, the relationship between learning theory concepts such as excitatory and inhibitory gradients and computational concepts such as pattern separation and completion remain unclear at the performance level, but are distinct at the process level. This distinction should help us frame experimental questions so that we can differentiate between the effects of these processes. In the realm of fear learning, we can tailor training to achieve various levels of acquisition, and determine how representations in the amygdala correspond to these excitatory gradients, and how they respond to discrimination training with a new stimulus. We can look for pattern completion at the population level in CA3 by labeling two conflicting representations and determining the circumstances where one or the other becomes activated and asserts its activity downstream in CA1. The literature abounds with studies involving the disruption of adult-born neurons and its effects on a given task, yet this strategy has yielded a complex set of seeming contradictions that has proven difficult to interpret. By designing experiments and interpreting results within a framework of clearly defined terms and strong theoretical predictions, we can begin to lay the groundwork for a new understanding of the role adult-born neurons play in learning.

## Conclusion

Our goal has not been to assign a single function to adult-born neurons in the context of learning. Rather, it has been to provide a framework that may be of use for future design of experiments and the discussion of results using clear terminology and concepts rooted both in learning theory and computational neuroscience. The lack of apparent consistency in results of manipulations of adult-born neurons is a good example of how vague terminology can hinder scientific progress.

Adult-born neurons seem to be involved in analyzing current stimuli, relating them to previous experiences, and determining the appropriate behavioral response. Whether the task is a generalization test, where excitation may generalize to similar stimuli, or discrimination learning, where repeated experience should alter response gradients to allow differentiation, adult-born neurons may alter the representation of these gradients, or how they change with experience, in order to affect outcome. During the test, the computational process of pattern separation would lead to these neurons representing the stimulus in a more distinct matter from other similar representations, while pattern completion would lead to using the stimulus cues to reactivate the most similar representation in order to guide behavior.

It is important to consider not only the effects of adult-born neurons on emotional learning, but how the various possible outcomes may serve the animal. During contextual fear discrimination learning, repeat experiences with the two stimuli give the animal the opportunity to update representations to respond differently, even though the default response to the two stimuli before learning would be generalization. This generalization default for similar stimuli is highly adaptive; animals that can use memory of a previous experience to guide current responding will have an advantage over those that treat each experience as novel, without making parallels between them. In learning about regularities of an environment, such as where food can be found and how to avoid predators, generalization conveys a starting point for appropriate behavior that is often correct. However, in particular instances where the standard or generalized response should not apply, it may take considerable effort or experience to create a differentiated representation that can specifically guide behavior in those circumstances.

The fact that adult born-neurons may alter the balance between similar or differential responding to related stimuli suggests new hypotheses. Perhaps adult-born neurons may alter the shape of excitatory gradients, affecting both generalization and differentiation. Or perhaps they alter the method of updating these gradients, either excitatory or inhibitory or both, leading to selective effects on differentiation. It seems that context and tone fear conditioning are powerful experimental tools that may be used to tease apart these effects. One avenue that may also provide insight is the use of the hybrid or transposition task designs, which have not been studied in the context of adult-born neurons. By altering the relative distance between trained stimuli and test stimuli, as well as the similarity of test stimuli to one or the other of the trained stimuli, experiments would be able to tease apart selective effects on excitatory and inhibitory gradients that may be difficult to discern during discrimination learning alone. Furthermore, as both hybrid and transposition designs often lead to choice of the novel stimulus at test, the neural correlates underlying the difference between sequential and concurrent discrimination learning may also offer insight into the role of adult-born neurons. The use of context fear to study peak shift is complicated by the multiple stimulus dimensions that make up a given context, but with careful experimental design and context manipulation, perhaps finally some light can be shed on the specific processes that adult-born neurons in the dentate gyrus uniquely mediate.

### Conflict of interest statement

The authors declare that the research was conducted in the absence of any commercial or financial relationships that could be construed as a potential conflict of interest.
